# A Systematic Review of the Impact of Intergenerational Learning on the Psychosocial Well-being of Primary School Children and Older Adults.

**DOI:** 10.1192/j.eurpsy.2024.703

**Published:** 2024-08-27

**Authors:** E. Tsiloni, E. Dragioti, M. Gouva, S. P. Vassilopoulos, M. Mentis

**Affiliations:** ^1^Department of Educational Sciences and Social Work, University of Patras, Patra; ^2^Research Laboratory Psychology of Patients, Families & Health Professionals, Department of Nursing, School of Health Sciences, University of Ioannina, Ioannina, Greece

## Abstract

**Introduction:**

In recent times, there has been a growing emphasis on the significance of fostering intergenerational learning and interaction. This involves individuals from diverse age groups engaging in purposeful and mutually beneficial activities aimed at enhancing their knowledge, skills, and values.

**Objectives:**

This systematic review was undertaken to explore the psychosocial consequences of intergenerational learning experiences among primary school-age children and older adults.

**Methods:**

In accordance with the PRISMA guidelines, a comprehensive review of both quantitative and qualitative data was conducted. Electronic databases such as PubMed, Scopus, and ERIC were meticulously searched up to July 26, 2022, using the following Population (P) - Exposure (E) - Outcome (O) criteria: primary school-age children and older adults (P), participation in intergenerational learning (E), and psychosocial effects (O). Additionally, we extensively scrutinized the reference lists of included datasets and pertinent review articles (Figure 1). To evaluate the quality of the eligible studies, we employed the Mixed Methods Appraisal Tool (MMAT). Data analysis was structured around a narrative synthesis approach.

**Results:**

A total of seventeen studies were deemed eligible for inclusion in this review. The findings regarding the psychosocial consequences of engaging in intergenerational activities for both children and older adults predominantly underscored positive improvements in their attitudes, well-being, happiness, and various other aspects of their social and psychological well-being, although certain methodological limitations were identified (Figure 2).

**Image:**

**Figure:**
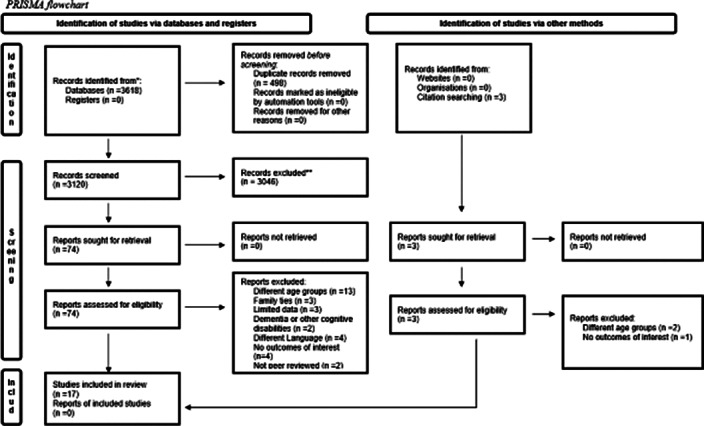

**Image 2:**

**Figure:**
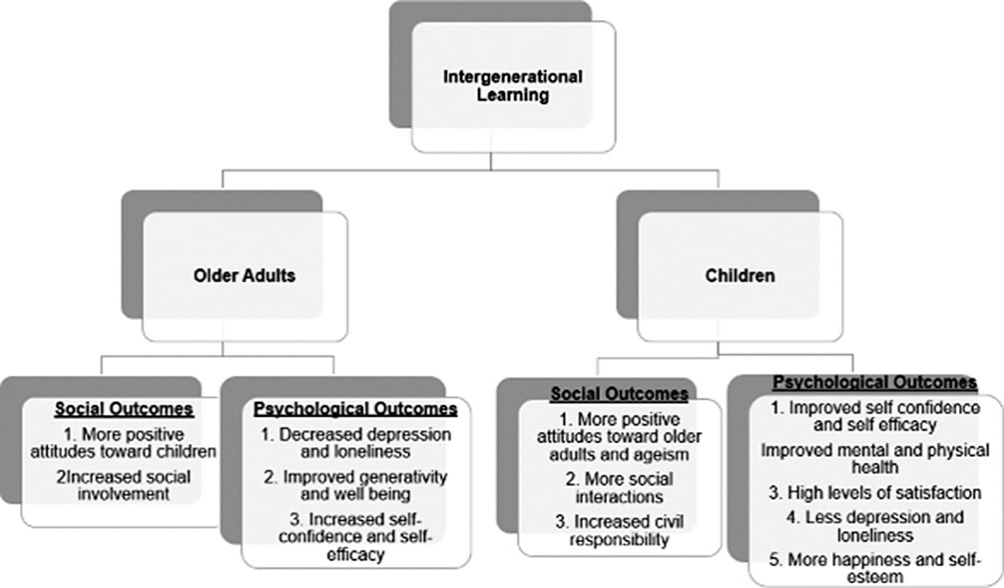

**Conclusions:**

Promoting intergenerational interactions and learning experiences holds promise as a means to enhance the overall quality of life and well-being for both younger and older members of our communities.

**Disclosure of Interest:**

None Declared

